# Differential Regulation of DNA Methylation at the CRMP2 Promoter Region Between the Hippocampus and Prefrontal Cortex in a CUMS Depression Model

**DOI:** 10.3389/fpsyt.2020.00141

**Published:** 2020-03-18

**Authors:** Dan Xiang, Jiawei Xiao, Siqi Sun, Linyan Fu, Lihua Yao, Gaohua Wang, Zhongchun Liu

**Affiliations:** ^1^Department of Psychiatry, Renmin Hospital of Wuhan University, Wuhan, China; ^2^Institute of Neuropsychiatry, Renmin Hospital, Wuhan University, Wuhan, China

**Keywords:** depression, CUMS, DNA methylation, CRMP2, neural plasticity

## Abstract

Current evidence supports the idea that neural plasticity is a potential cause of depression. Abundant studies indicate that CRMP2 has important roles in neural plasticity. Moreover, CRMP2 may contribute to the etiology of depression. However, the regulatory mechanisms underlying the role of CRMP2 remain unclear. DNA methylation alteration is generally acknowledged to be involved in the development of depression. The aim of this study was to explore the relationship between the expression and DNA methylation of CRMP2 in the hippocampus and prefrontal cortex of a rat depression model. Chronic unpredictable mild stress (CUMS) was used to establish a rat depression model, and body weight and behavioral tests were used to evaluate the effects of stress. Real-time PCR and Western blotting were used to test CRMP2 mRNA and protein expression, respectively, in the hippocampus and prefrontal cortex of rats. DNA methylation levels of the CRMP2 promoter were analyzed by bisulfite sequencing PCR (BSP). CUMS caused depressive-like behavior in rats, as evidenced by: decreased body weight and sucrose preference rate; decreases in the total distance traveled, rearing frequency, velocity, and duration in the center in the open field test (OFT); and prolonged immobility in the forced swimming test (FST). CRMP2 mRNA and protein expression in the hippocampus and prefrontal cortex were significantly decreased in the CUMS group compared with the control group. The levels of CRMP2 promoter DNA methylation in the hippocampus of the CUMS group were significantly higher than those of the control group, while these changes were not observed in the prefrontal cortex of CUMS rats. Our data provide evidence that altered expression of CRMP2 in the hippocampus and prefrontal cortex is associated with the pathogenesis of depression. Moreover, the results also suggest regional differences in the regulation of DNA methylation in the CRMP2 promoter between the hippocampus and prefrontal cortex during the development of depression.

## Introduction

Depression is a highly prevalent psychiatric disorder characterized by low moods, anhedonia, and loss of interest or pleasure, and is associated with high levels of morbidity and mortality (1). Depression has been diagnosed in more than 300 million people at present, with an increase of more than 18% between 2005 and 2015 (2). It will be a major contributor to healthcare costs and is projected to be the leading cause of disease burden in middle- and high- income countries by the year 2030 (3). These data regarding depression suggest that it is very common, with an enormous burden for society and for patients. Although some standard pharmacological and non-pharmacologic therapies for depression are effective, the etiology of depression remains unclear. Therefore, a new understanding of the pathogenesis of depression should be explored.

The collapsin response mediator protein (CRMP) family comprises five homologous proteins (CRMP 1-5). CRMP1,2,3, and 4 display 75% homology with each other, while CRMP5 shares only 50% homology with the other 4 homologs (4). Several studies have revealed that CRMPs play important roles in various stages of neurodevelopment and are involved in the pathogenesis of neuropsychiatric diseases (5, 6). Among the five CRMP isoforms, only CRMP2 is highly expressed in adult neurons. CRMP2 protein, also identified as dihydropyrimidinase-like protein 2 (DPYSL 2), is highly expressed in the mammalian-nervous system, including the hippocampus, olfactory bulb, cerebellum, and cortex (7, 8). CRMP2 is a microtubule-binding protein that regulates cytoskeletal dynamics, vesicle trafficking, and synaptic transmission in neurons (9, 10). *In vitro*, CRMP2 has been implicated in regulating several aspects of neuronal function and differentiation, including dendritic and axonal growth, kinesin-dependent axonal transport, synaptic physiology, and neurotransmitter release (11, 12). Additionally, studies have further suggested that CRMP2 regulates neuronal polarity and migration (13). Consistent with the role of CRMP2 in neuronal functions, it has been associated with neuropsychiatric diseases including Alzheimer's disease, schizophrenia, and depression. In patients with, and genetic mouse models of, Alzheimer's disease, increased phosphorylated CRMP2 has been detected in brain tissue and is associated with neurofibrillary tangles (14). Multiple proteome-wide analyses have suggested significant changes in CRMP2 expression in the brains of schizophrenia patients and lower expression of CRMP2 in the postmortem brains of schizophrenia patients (15). Proteomic technology has revealed that CRMP2 levels are decreased in the frontal cortex brains of patients with depression (16). Moreover, Proteomic methodology also showed that CRMP2 was downregulated in the hippocampus in stressed rats (17). The proteomic analysis of rat hippocampus and frontal cortex after chronic treatment with an SSRI antidepressant detected an increase in CRMP2 expression (18). However, the potential role of CRMP2 in the pathogenesis of depression remains unknown.

Depression is a multifactorial illness that is guided by the interaction between genetic susceptibility and environmental stimuli. Several animal and human studies have suggested that epigenetic mechanisms can modulate DNA transcriptional functionality in response to the environment and might play a role in the processes that contribute to the pathophysiology of depression (19, 20). Epigenetic modifications include DNA methylation, histone modification, chromatin conformational changes, and non-coding RNA, and DNA methylation is the most studied epigenetic modification to date. DNA methylation is a process catalyzed by DNA methyltransferase enzymes (DNMTs). According to the roles and specificities of DNMTs, they are divided into maintenance DNMTs, such as DNMT1, and *de novo* DNMTs, which include DNMT3a and DNMT3b (21, 22). *De novo* DNMTs are responsible for the binding of a methyl group onto the C5 position of the cytosines in cytosine-phosphate-guanine dinucleotides (CpG) sites. Maintaining DNA methylation in the context of replication and cell division is accomplished primarily by DNMT1 (23, 24). DNA methylation occurs in regions with a greater density of CpG sites and is frequently enriched in promoter regions, or so-called CpG islands. DNA methylation is the most stable form of epigenetic alteration, and several studies have suggested that aberrant DNA methylation is associated with depression (25, 26). According to several preclinical animal models, chronic stress appears to alter DNA methylation and has subsequent effects on gene expression. In a rodent study, Weaver et al. have found that offspring with neglectful mothers showed changes in DNA methylation levels of glucocorticoid receptor (GR) in hippocampal tissue (27). The study by Roth et al. found that caregiver ill-treatment was associated with increased methylation of brain-derived neurotrophic factor (BDNF) in the prefrontal cortex of rodents (28). With the rapid development of technological advances in the field of genomics, genome-wide association studies (GWASs) were performed in patients with depression. In a GWAS study, 224 candidate regions with DNA methylation differences in highly enriched regions were identified as neuronal development and growth genes (29). Furthermore, DNMTs play a role in depression, and DNMT 3 was expressed at higher levels in the nucleus accumbens of mice models of depression than in control mice, and DNA methyltransferase inhibitors have been reported to play an antidepressant effect (30). DNA methylation levels are subject to changes in responses to environmental stimuli, and they provide a potential mechanism to account for the gene-environment interactions in the pathophysiology of depression. Together, abundant studies indicate that CRMP2 may contribute to the pathogenesis of depression. However, the underlying mechanisms remain unclear. We hypothesized that abnormal DNA methylation would affect the expression of CRMP2 in stressed rats. In the present study, we analyzed the DNA methylation level of CRMP2 promoter in stressed rats to evaluate the contribution of CRMP2 promoter methylation to depression.

Depression is often precipitated or exacerbated by chronic stressful events, and chronic stress is usually used to induce behavior models of depression. In the chronic unpredictable mild stress (CUMS) procedure, exposure of animals to different kinds of mild stress every day produced a longer lasting depression-like behavior. The CUMS model recapitulates many of the core behavioral characteristics of depression, therefore, the model has been widely used for investigating the pathophysiology of depression and its associated therapeutic interventions (31). In our study, we used CUMS to establish a rat model of depression and explored the effects on depression-like behavior using the sucrose preference test (SPT), open field test (OFT), and forced swimming test (FST). Furthermore, the hippocampus is part of the limbic structures, which are associated with emotional responses, and it is the most commonly studied brain region in depression research. The prefrontal cortex, as a significant nerve center of emotional regulation in the brain, is also associated with depression. From a neural plasticity point of view, the hippocampus and prefrontal cortex plasticity is widely considered to play a significant role in the onset and development of depression (32). Thus, we measured CRMP2 expression and DNA methylation levels in the hippocampus and prefrontal cortex of rats with the aim of exploring the underlying mechanisms of the pathological processes of depression.

## Methods

### Animals

Male Sprague-Dawley (SD) rats weighing 180–200 g were purchased from the Company of Experimental Animals of Hunan Slack King (Hunan, China). Before the experiment, the rats were adapted to the laboratory conditions for 1 week. The rats were maintained at 22 ± 2°C with a 12 h light/12 h dark schedule with free access to food and water. All of the experimental procedures were in accordance with the guidelines of the P.R. China legislation on the ethical care and use of laboratory animals and approved by the Institutional Animals Care Committee of Renmin Hospital of Wuhan University.

### Experimental Design

**Figure 1:**
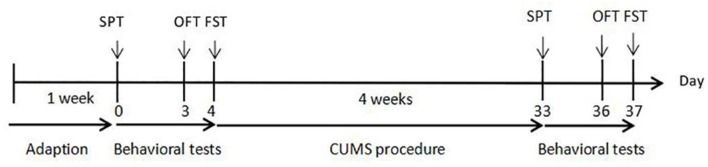
Experimental procedures. CUMS: 28 days of chronic unpredicted mild stress. SPT, sucrose preference test on day 0 and day 33; OFT, open field test on day 3 and day 36; FST, forced swimming test on day 4 and day 37.

Before application of the CUMS procedure, we recorded the body weight of, and performed behavioral tests on, a group of rats, the results of which were used to remove outliers. Then, the male rats were randomly divided into two groups: the control group, and the CUMS group (*n* = 15 per group). Rats in the CUMS group were exposed to a series of CUMS procedures for 4 weeks. The control group did not accept any CUMS stimuli throughout the procedure. Body weight was measured and behavioral tests were performed to detect the effects of CUMS. The experimental procedures are shown in Figure 1.

### CUMS Procedure

The CUMS protocol used in the experiment was adapted from the former published papers (33), with slight modifications. The protocol included: cold water swimming at 4°C for 5 min; heat stress at 45°C for 5 min; water deprivation for 24 h; food deprivation for 24 h; tail clamp for 1 min; tilted cage (45°) for 24 h; and exposure to damp sawdust for 24 h. Animals were given one type of stimulus at a random time every day in order to be completely unpredictable to the rats.

### Body Weight and Behavioral Tests

Body weight measurements and behavioral tests were conducted before the CUMS procedure began and after the end of the 4 week CUMS procedure. The rats were subjected to the behavioral tests in the following order: SPT, OFT, and FST.

#### Sucrose Preference Test (SPT)

The SPT is a common method used to quantify anhedonia, an important symptom of depression. Before the test, all rats were trained to consume a 1% sucrose solution. After the training session, the rats were deprived of water and food for 24 h. For the test, the rats were given free access to one bottle with 100 ml of sucrose solution and another bottle with 100 ml of pure water. The solution weights were recorded at the beginning and the end of the 24 h test. The sucrose preference percentage was calculated as the following: Sucrose preference rate (%) = sucrose consumption / total solution consumption × 100%.

#### Open Field Test (OFT)

The OFT was performed to assess general locomotor activity and exploratory behavior in the rats. The open field apparatus consisted of a rectangular cage (100 cm × 100 cm × 35 cm) and a video tracking system (Ethovision XT 11.5). The test was performed in a quiet environment. Each rat was placed at the center of the open-field and observed for 5 min. The number of times the rat reared, total distance moved, average velocity, and duration in the center were recorded. Between each rat test, the cage was thoroughly cleaned with 75% alcohol to avoid potential infections.

### Forced Swimming Test (FST)

The FST is typically used to assess classical depressive-like behavior/despair behavior in animals. According to the previously published protocol, the swimming procedure consists of placing the rats into a cylindrical tank (30 cm diameter × 40 cm height) filled with water at 25°C to a depth of 28 cm. The rats were submitted to forced swimming for 15 min (pre-test), and the next day, the rats were put back into the water and underwent the FST. Each rat was forced to swim for 5 min, and the immobility time was recorded while the rat was floating in the water without struggling or only using minimal movements to keep its head above the water. After the forced swimming, the rats were gently dried and placed back into their cages.

### Sample Collection

After the final behavioral tests, the rats were euthanized under general anesthesia. Whole brains were removed on ice, and the whole hippocampus and prefrontal cortex from both hemispheres were carefully isolated. The samples were stored at −80°C until assays.

### RNA Extraction and RT-PCR

The Trizol extraction reagent (Ambion, 15596-026), was used to extract total RNA from the hippocampus and prefrontal cortex according to the manufacturer's instruction. The concentration and purity of the RNA samples were determined by a spectrophotometer (Thermo; NanoDrop 2000) at 260 nm. Three micrograms of RNA were reverse transcribed into cDNA using an RT-PCR kit (Thermo; K1622). Real-time PCR specific primers for rat CRMP2 and GAPDH were designed using Primer Premier 5 software. The primer used in this study were as follows: CRMP2 forward: 5′-ACTCGCTTCCAGATGCCAGAC-3′; CRMP2 reverse: 5′-GTGCCACTCCGTGATGTCCA-3′; GAPDH forward: 5′-CTGGAGAAACCTGCCAAGTATG-3′; GAPDH reverse: 5′-GGTGGAAGAATGGGAGTTGCT-3′. The cDNA was subsequently amplified by PCR using SYBR Premix Ex Taq^TM^ II (Takara; RR820A) and carried out in triplicate using a CFX96 real-time PCR detection system (Bio-Rad). The reaction program for the PCR was as follows: 1 cycle of 95°C for 30 s, 38 cycles of 95°C for 5 s, 60°C for 30 s, and extension at 72°C for 30 s. GAPDH was chosen as an internal control for normalization. Triplicate PCR amplifications were performed for each sample. The expression of CRMP2 mRNA was analyzed by the 2^−ΔΔCT^ method.

### Protein Extraction and Western Blot

Total proteins of the hippocampus and prefrontal cortex brain tissue were extracted from each group with 1% PMSF in 1 mL of ice-cold RIPA buffer (Beyotime, P0013B), with added protease inhibitor cocktail. The tissues were suspended in RIPA buffer, homogenized on ice using an ultrasound homogenizer, and lysed on ice for 30 min. After homogenizing and centrifuging at 12,000 rpm at 4°C for 15 min, the supernatant proteins were preserved. The BCA kit (Thermo, 23228) was used to determine the concentration of protein. All the normalized protein samples were separated by 12% polyacrylamide gel electrophoresis using a constant voltage. Next, the proteins were transferred to polyvinylidene difluoride (PVDF) membranes (Merck Millipore, ISEQ00010), blocked with 5% non-fat milk, and incubated with primary antibodies overnight at 4°C, including anti-CRMP2 antibody (Abcam, EPR7792, 1:10000 dilution) and anti-GAPDH antibody (Abcam, ab9485, 1:5000 dilution). The PVDF membranes were washed with Tris-buffered saline containing Tween-20 (TBST) 3 times for 10 min each on the following day. Then, the membranes were incubated with secondary antibodies (Beyotime, A0208, 1: 5000) at room temperature for 2 h, and again washed with TBST 3 times for 10 min each. Protein bands were detected using an ECL chemiluminescent detection kit (Beyotime, P0018S). Individual band intensity was quantified using ImageJ software, and GAPDH was used as an internal control to analyze the relative CRMP2 protein quantity. Each brain sample was run three times, and at least three independent sample pairs were used for each statistical analysis.

### Bisulfite Sequencing PCR (BSP)

Genomic DNA from tissue from the hippocampus and prefrontal cortex was isolated using a genomic DNA extraction kit (Tiangen, DP304) according to the manufacturer's instructions. DNA was subjected to bisulfite modification using an EpiTect Bisulfite Kit (Qiagen, 59104). Bisulfite treatment induces the conversion of unmethylated CG sites to uracil-guanine pairs (UG) with no effect on methylated sites. The promoter of the CRMP2 gene is generally considered to be a 2,000 bp sequence upstream of the transcription start site. The primer used to amplify the bisulfite treated DNA was designed with the MethPrimer software. The BSP primer was as follows: F: 5′-TTTGTATTGTAGATGAAGTATTTGGG-3′; R: 5′- AACAATAAAAACCTTAATTCCAATC-3′. The PCR was performed under the following conditions: 95°C for 30 s, 40 cycles of 95°C for 5 s, 50°C for 30 s, and 72°C for 30 s. PCR products were assessed by 2% agarose gel electrophoresis and purified by gel extraction using a gel extraction kit (Cwbio, CW2302M). The purified products were inserted into a pEASY-T1 cloning vector (TransGen, CT101-01) and transformed into *E. coli* DH5αfollowing the manufacturer's instructions. A minimum of 10 positive clones were chosen from LB agar plates for each sample and sequenced by the Wuhan Tianyi Huiyuan Company.

### Statistical Analysis

All data are presented as the means ± standard error of the mean (SEM). Statistical analysis was performed using SPSS 20.0 software. The raw data were collected by the investigators who were blinded to the grouping of the animals. All the data were normally distributed according to the Shapiro-Wilk test. Differences between the two groups were analyzed by Student's *t*-test. Value for *P* < 0.05 were considered to be statistically significant.

## Results

### Body Weight and Behavioral Tests

**Figure 2:**
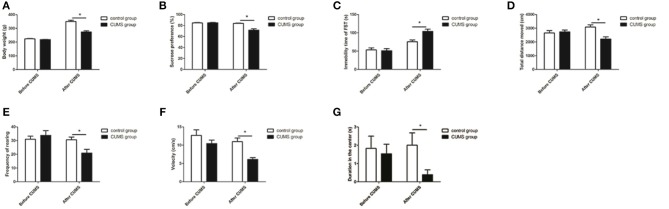
Body weight and behavioral tests. Effects of CUMS on body weight **(A)** and sucrose preference rate in the SPT **(B)**; immobility time in the FST **(C)**; total distance traveled **(D)**, rearing frequency **(E)**, velocity **(F)**, and duration in the center **(G)** in the OFT. *P < 0.05 vs. the control group.

The body weight and behavioral tests were conducted before the CUMS procedure began and at the end of 4 weeks of CUMS. The behavioral tests included the SPT, OFT, and FST. Before the CUMS procedures, the body weight of the rats in each group did not have a significant difference, and there was also no significant difference in sucrose preference rate, immobility time in the FST, and total distance traveled, rearing frequency, velocity, and duration in the center in the OFT. After 4 weeks of CUMS, the CUMS group showed: a lower body weight [*t*_28_ = 6.466, *P* < 0.05, Figure 2A] and sucrose preference rate [*t*_28_ = 5.271, *P* < 0.05, Figure 2B]; lower total distance traveled [*t*_28_ = 3.779, *P* < 0.05, Figure 2D], rearing frequency [*t*_28_ = 2.914, *P* < 0.05, Figure 2E], velocity [*t*_28_ = 4.433, *P* < 0.05, Figure 2F], and duration in the center [*t*_28_ = 2.241, *P* < 0.05, Figure 2G] in the OFT; and increased immobility time [*t*_28_ = −3.763, *P* < 0.05, Figure 2C] in the FST compared with the control group.

### CRMP2 mRNA Expression Level

**Figure 3:**
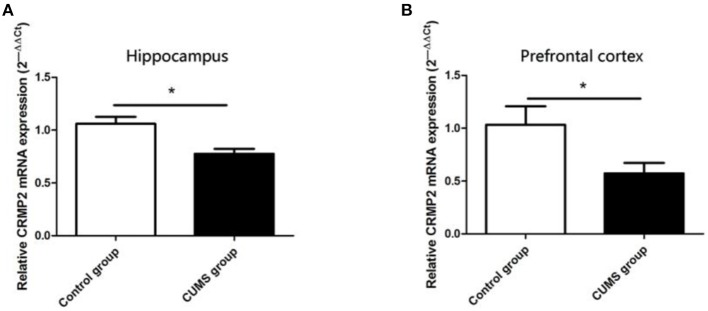
Expression of CRMP2 mRNA in the control and CUMS groups. Expression of CRMP2 mRNA in the hippocampus **(A)** and prefrontal cortex **(B)** were measured by RT-PCR. *P < 0.05 vs. the control group.

The CRMP2 mRNA expression level was measured using RT-PCR. As shown in Figure 3, chronic stress significantly decreased the CRMP2 mRNA expression level in both the hippocampus [*t*_8_ = 3.500, *P* < 0.05, Figure 3A] and prefrontal cortex [*t*_8_ = 2.283, *P* < 0.05, Figure 3B] in the CUMS group compared with the control group.

### CRMP2 Protein Expression Level

**Figure 4:**
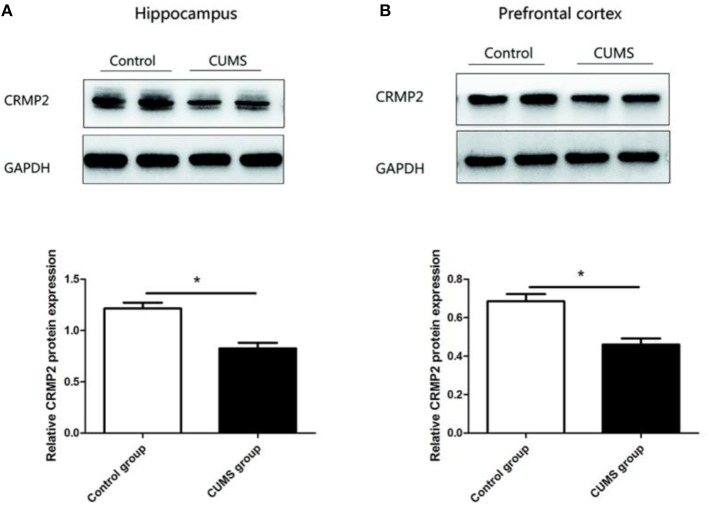
Expression of CRMP2 protein in the control and CUMS groups. Western blot analysis showed the expression of CRMP2 protein in the hippocampus **(A)** and prefrontal cortex **(B)**. *P < 0.05 vs. the control group.

Similar observations were observed in CRMP2 protein expression in the hippocampus and prefrontal cortex of the CUMS group. Compared with the control group, the CRMP2 protein expression level was significantly downregulated in the hippocampus [*t*_8_ = 6.507, *P* < 0.05, Figure 4A] and prefrontal cortex [*t*_8_ = 4.929, *P* < 0.05, Figure 4B] of the CUMS group.

### DNA Methylation Level in CRMP2 Promoter Region

**Figure 5:**
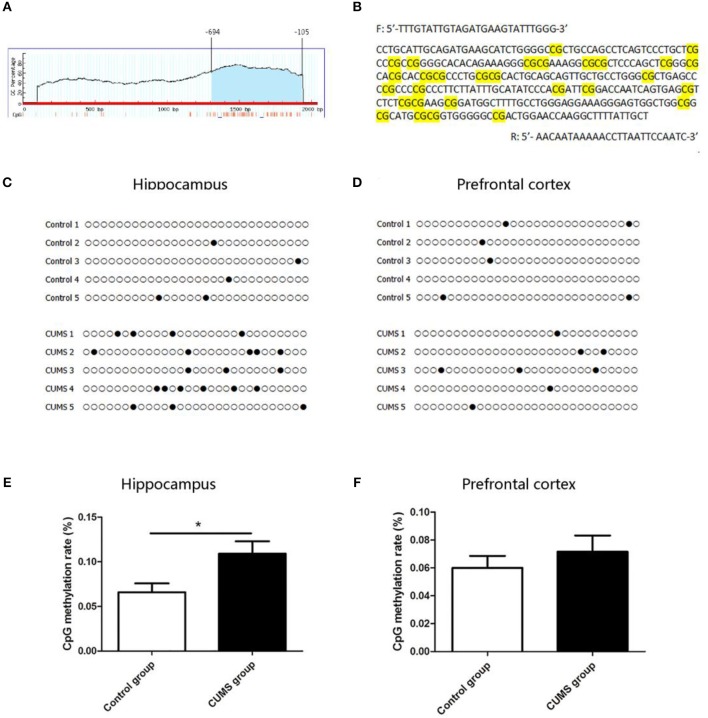
DNA methylation level in CRMP2 promoter region in the control and CUMS groups. One CpG island is located from nucleotides −694 to −105 with 29 CpG sites in the promoter region of the CRMP2 gene, short vertical lines indicate CpG dinucleotides **(A)**; using Methyl Primer software, BSP primer targeted the CpG islands in the promoter region of CRMP2 was designed, and the CpG dinucleotides are highlighted **(B)**; the methylation levels of the promoter region of the CRMP2 gene in the hippocampus **(C,E)** and prefrontal cortex **(D,F)**, black circles represent methylated CpG sites, and white circles represent unmethylated CpG sites. *P < 0.05 vs. the control group.

The CRMP2 gene is located on 15p12 of rat chromosome. The promoter of the CRMP2 gene is generally considered as a 2,000 bp sequence upstream of the transcription start site. The CpG island of the CRMP2 promoter was predicted using MethPrimer online software. We found that there was 1 CpG island located from −694 to −105 with 29 CpG sites in the promoter region of the CRMP2 gene, as shown in Figure 5A. Figure 5B shows that the BSP primer targeted the CpG island in the promoter region of the CRMP2 gene, and the CG dinucleotides were highlighted. DNA methylation status of the CRMP2 promoter were analyzed by BSP. Treating genomic DNA with bisulfite resulted in the conversion of all unmethylated cytosine to uracil, and the methylated cytosine remained unchanged. The methylation status of the promoter region of the CRMP2 gene is shown in Figures 5C,D, and each row indicates the sequence of an individual clone. Black circles represent methylated CpG sites and white circles represent unmethylated CpG sites. Our data demonstrate that the DNA methylation level at the CRMP2 promoter region in the hippocampus of the CUMS group increased compared with that in the control group. The methylation rate was calculated as the number of CpG methylated sites/number of CpG sites. The DNA methylation rate of the CRMP2 promoter in the hippocampus of the control rats was 6.6 ± 0.9%. After 4 weeks of CUMS, the DNA methylation rate (10.9 ± 1.3%) at the CRMP2 promoter region in the hippocampus of the CUMS group significantly increased relative to that in the control group [*t*_8_ = −2.514, *P* < 0.05, Figure 5E]. However, no differences in CRMP2 promoter DNA methylation in the prefrontal cortex were observed (6.0 ± 0.8% for the control group; 7.1 ± 1.2% for the CUMS group) [*t*_8_ = −0.799, *P* > 0.05, Figure 5F].

## Discussion

In our study, depression models were successfully induced by 4 weeks of CUMS. As expected, the CUMS rats exhibited marked decreases in: body weight; sucrose preference rate; total distance traveled, rearing frequency, velocity, and duration in the center of the OFT; and increased immobility time in the FST compared to control rats. A lower body weight in the CUMS rats indicated the progression of depression-like behavior since weight loss is considered one of the symptoms of depression (34). Our CUMS rats exhibited a decreased consumption of the sucrose solution in the SPT, suggesting anhedonia in rats(a core symptom of depression). Immobility time in the FST is believed to mirror despair present in human depression, and we replicated the finding that CUMS can induce an increased immobility time in the FST, consistent with previous reports (35, 36). Additionally, depression-like behavior was confirmed by the reduction of total distance traveled, rearing frequency, and velocity observed during the OFT, which has been widely used to assess the spontaneous general activity and explorative behavior of rats. Moreover, the duration in the center of OFT has been used to examine anxiety-like behavior in rodents. Increased depressive-like and anxiety-like behavior in OFT in CUMS rats were observed in our study.

In recent years, there has been increasing interest in the correlation between depression and neural plasticity. The neural plasticity hypothesis proposes the theory that dysfunction of neural plasticity is a critical pathogenesis of depression (37). Synaptic plasticity is one of the most fundamental and important functions of the brain, and stress decreases synaptic plasticity in the hippocampus and prefrontal cortex (38). Additionally, it has been widely reported that limbic and cortical region atrophy, including decreased volumes of the hippocampus and prefrontal cortex, can be found in depression patients (39, 40). Some studies have suggested that volumetric reductions could be due to changes in neuropil, glial number, and structural dendrite alterations (41). Moreover, the neurogenic hypothesis of depression emphasizes the contribution of hippocampal neurogenesis to the pathogenesis of depression (42). These studies suggest that dysregulation of synaptic and neuronal plasticity may contribute to the pathophysiology of depression. CRMP2 is the most highly expressed CRMP in the adult brain, and it is mainly expressed in brain areas that retain plasticity or neurogenesis, such as the hippocampus, cerebellum, olfactory bulb, and several cortical areas. CRMP2 is known to regulate axonal growth and branching as a partner of the tubulin heterodimer, and plays a role in synaptic plasticity (43). CRMP2 can be phosphorylated by Ca^2+^/calmodulin-dependent protein kinase II at postsynaptic densities, suggesting that CRMP2 may be involved in synaptic plasticity (44). Several genomics and proteomics studies have associated transcription and translation changes of CRMP2 with depression. CRMP2 knockout mice exhibited structural deficits in neurons that would affect synaptic plasticity (45). Proteomic technology has revealed that CRMP2 levels are decreased in the hippocampus and frontal cortex brains of stressed rats (46). Chronic treatment with antidepressants, venlafaxine, and fluoxetine, increased the expression of CRMP2 in the hippocampus (47). Together with the previous findings, our data suggest that 4 weeks of the CUMS procedure induced a reduction of CRMP2 mRNA and protein expression in the hippocampus and prefrontal cortex, and these changes were accompanied by depressive-like behaviors. These data suggest that CRMP2 might be a key player in the development of depressive-like behaviors induced by CUMS. Additionally, consistent with previous reports, our study found that CRMP2 mRNA and protein expression in the hippocampus and prefrontal cortex were significantly decreased in stressed rats. The results suggest that the changes of CRMP2 expression is similar in the hippocampus and prefrontal cortex in depression. However, increased levels CRMP2 were observed in the anterior cingulate cortex of depression (48). These findings suggest that alteration of CRMP2 expression can elicit depressive behavior depending upon the specific brain regions.

Emerging evidence suggests that early exposure to stress is associated with increased vulnerability to depression. DNA methylation may be involved in vulnerability to stress and depression and likely modulates synaptic and structural plasticity (49). Environmental factors induce changes in DNA methylation levels at the promoters of specific genes, and the effectiveness of antidepressants may be attributed to the tendency of DNA methylation to change (50, 51). Candidate genes of DNA methylation, such as NR3C1, SLC6A4, and BDNF, have been regarded as potential biomarkers of depression. The NR3C1 gene, encoding the GR, is an essential factor for the effective functioning of the hypothalamic pituitary adrenal (HPA) axis (52). The gene SLC6A4 that encodes the serotonin transporter (5-HTT) is involved in the regulation of serotonergic signaling (53). Moreover, among the potential candidates is the gene coding for BDNF, which belongs to the neurotrophin family, and promotes the proliferation, differentiation, and survival of neurons and is crucial for neural plasticity and cognitive function (54). The processes of the HPA axis, serotonergic system, and neurotrophic signaling play a key role in the pathogenesis of depression, and DNA methylation may serve as a critical regulatory mechanism.

The effect of DNA methylation on gene expression is known to be a function of the methylation site. Methylation in CpG islands of gene promoter regions has been researched and the popular notion is that methylation leads to a repression of transcription. The promoter region of the CRMP2 gene contains one CpG island (from −694 to −105) with 29 CpG sites. In this study, we mainly focused on the methylation status of 29 CpG sites in the CpG island of the CRMP2 gene promoter. This is the first study to assess CRMP2 mRNA and protein expression and DNA methylation levels in hippocampal and prefrontal cortex tissues. The aim of this study was to explain the relationship between CRMP2 expression and DNA methylation status in the promoter region of the CRMP2 gene. The results showed that 6.6 ± 0.9% of CpG at the CpG island in the CRMP2 promoter region exhibited methylated status in the hippocampus of the control group. The DNA methylation rate of the CpG island in the CRMP2 promoter region from the prefrontal cortex of the control group was 6.0 ± 0.8%. After administration of CUMS, the methylation rate at the CpG island in the CRMP2 promoter region increased significantly in the hippocampus compared to that of the control group. However, this change was not observed in the prefrontal cortex of CUMS rats. These data suggest that DNA methylation in the CRMP2 promoter is a potential regulatory mechanism for stress induced depressive-like behaviors. These results also provide evidence for regional differences in the regulation of DNA methylation in the CRMP2 promoter between the hippocampus and prefrontal cortex during stress.

To gain insight into the potential correlations between DNA methylation and gene expression, we must consider the distribution of CpG dinucleotides across the genome. Evidence has suggested that the methylation of CpG islands in promoter region plays an important role in the regulation of gene expression. When DNA is methylated, chromosomes become stabilized, with decreased activity and reduced gene expression (55). However, studies have shown a positive correlation between gene body methylation and gene expression, and this needs to be further confirmed (56). In our study, we mainly detected the level of DNA methylation in the CRMP2 promoter region. We demonstrated that chronic stress decreases CRMP2 expression in the hippocampus and that the DNA methylation levels in the CRMP2 promoter were increased in stressed rats compared with control rats. Based on these results, we propose that DNA methylation is responsible for the changes in CRMP2 expression in the hippocampus of stressed rats. Additionally, we also found decreased levels of CRMP2 in the prefrontal cortex of the stressed group compared to those in the control group, while we did not find differences in CRMP2 methylation levels in the prefrontal cortex between the groups. One possible explanation for this is that the observed expression changes may be related to other factors, such as the regulation of other transcription factors and other epigenetic mechanisms, such as histone modification and non-coding RNA silencing.

Recent data suggested that changes in DNA methylation can be both a cause and consequence of transcription factor binding and may also be involved in stabilizing regulatory states (57). We try to predict transcription factor binding sites with TRANSFAC. The result showed that there were 13 transcription factor binding sites of multiple transcription factors (such as SP1, NFYA, MafB, etc.) in CpG island, and some transcription factors have multiple binding sites. It is suggested that the CRMP2 gene may be regulated by multiple transcription factors. Moreover, CpG dinucleotide is contained in the binding sites of multiple transcription factors. Therefore, the abnormal methylation in the CpG island may interfere with the binding of transcription factors and regulate the expression of CRMP2. This conclusion needs to be verified by further experiments.

One limitation of this study is that we only detected the status of DNA methylation at the CRMP2 promoter region in the hippocampus and prefrontal cortex of rats, but did not verify our findings *in vitro* assays. An *in-vitro* cell model will be established in future studies to support our findings in the present study. Another limitation is that we only used male rats in this study and there were only five samples in each group for every analysis. The sample size of this study is not large. It is necessary to elucidate whether the CRMP2 gene is sex-specific in depression and to further verify our finding in future large-scale clinical studies.

## Conclusion

In conclusion, epigenetic changes, such as changes in DNA methylation following exposure to stress, provide a potential mechanism for the development of depression. In this study, the results showed that CUMS induced a decrease in CRMP2 expression in the hippocampus, which may correlate with a significant increase in DNA methylation levels of the CRMP2 promoter region. However, the decreased CRMP2 expression in the prefrontal cortex induced by CUMS was not related to DNA methylation of the CRMP2 promoter. These findings suggest a possible link between the long-term effect of chronic stress and changes in DNA methylation of the CRMP2 promoter and provide novel insight to aid in the understanding of the etiology and mechanisms underlying depression.

## Data Availability Statement

The sequences of DNA methylation in CRMP2 promoter were deposited in GenBank, and the GenBank accession number is: Bankit 2312830.

## Ethics Statement

The animal study was reviewed and approved by the Institutional Animals Care Committee of Renmin Hospital of Wuhan University.

## Author Contributions

GW and ZL designed and supervised the study and revised the manuscript. DX carried out the experimental procedures and analyzed the data. DX interpreted results of experiments and drafted the manuscript. All authors provided feedback on the manuscript.

### Conflict of Interest

The authors declare that the research was conducted in the absence of any commercial or financial relationships that could be construed as a potential conflict of interest.
